# Glucocorticoid receptor mRNA and protein isoform alterations in the orbitofrontal cortex in schizophrenia and bipolar disorder

**DOI:** 10.1186/1471-244X-12-84

**Published:** 2012-07-20

**Authors:** Duncan Sinclair, Maree J Webster, Janice M Fullerton, Cynthia Shannon Weickert

**Affiliations:** 1 Schizophrenia Research Institute, Liverpool St, Darlinghurst, NSW, 2011, Australia; 2 Neuroscience Research Australia, Hospital Rd, Randwick, NSW, 2031, Australia; 3 School of Psychiatry, Faculty of Medicine, University of New South Wales, Sydney, NSW, 2052, Australia; 4 Stanley Medical Research Institute, Laboratory of Brain Research, 9800 Medical Center Drive, Rockville, MD, 20850, USA; 5 School of Medical Sciences, Faculty of Medicine, University of New South Wales, Sydney, NSW, 2052, Australia

## Abstract

**Background:**

The orbitofrontal cortex (OFC) may play a role in the pathogenesis of psychiatric illnesses such as bipolar disorder and schizophrenia, in which hypothalamic-pituitary-adrenal (HPA) axis abnormalities are observed and stress has been implicated. A critical component of the HPA axis which mediates cellular stress responses in the OFC, and has been implicated in psychiatric illness, is the glucocorticoid receptor (GR).

**Methods:**

In the lateral OFC, we employed quantitative real-time PCR and western blotting to investigate GR mRNA and protein expression in 34 bipolar disorder cases, 35 schizophrenia cases and 35 controls. Genotype data for eleven GR gene (NR3C1) polymorphisms was also used to explore possible effects of NR3C1 sequence variation on GR mRNA and protein expression in the lateral OFC.

**Results:**

We found no diagnostic differences in pan GR, GR-1C or GR-1F mRNA expression. However, the GR-1B mRNA transcript variant was decreased (14.3%) in bipolar disorder cases relative to controls (*p* < 0.05), while GR-1H mRNA was decreased (22.0%) in schizophrenia cases relative to controls (*p* < 0.005). By western blotting, there were significant increases in abundance of a truncated GRα isoform, putative GRα-D1, in bipolar disorder (56.1%, *p* < 0.005) and schizophrenia (31.5% *p* < 0.05). Using genotype data for eleven NR3C1 polymorphisms, we found no evidence of effects of NR3C1 genotype on GR mRNA or GRα protein expression in the OFC.

**Conclusions:**

These findings reveal selective abnormalities of GR mRNA expression in the lateral OFC in psychiatric illness, which are more specific and may be less influenced by NR3C1 genotype than those of the dorsolateral prefrontal cortex reported previously. Our results suggest that the GRα-D1 protein isoform may be up-regulated widely across the frontal cortex in psychiatric illness.

## Background

The orbitofrontal cortex (OFC) is a critical associative area of the human cortex, which plays a central role in emotion processing, reward encoding and goal directed learning [1-3]. It has been implicated in the pathogenesis of a number of psychiatric disorders including bipolar disorder, schizophrenia and major depression [4,5].

The OFC includes regions in medial and lateral orbital cortical areas, including Brodmann’s areas (BA) 10–14, 25 and 47 [6,7]. These regions form two connective networks, the medial and lateral (orbital) OFC networks, which have few connections between them, receive different inputs and project to different cortical and subcortical areas [4,6]. The lateral OFC network, consisting of central, lateral and caudal areas of orbital cortex, is characterised by inputs from sensory cortical areas, such as the olfactory cortex and visual areas in the inferior temporal cortex [8]. It integrates multimodal sensory information and is involved in reward encoding and the evaluation of punishment [4,7]. It does not have robust connections to/from limbic regions, the dorsolateral prefrontal cortex (DLPFC) or visceral control centres such as the hypothalamus [8-10]. In contrast, the medial OFC network consists of areas along the medial edge of the orbital cortex, as well on the ventromedial surface of the frontal lobe [11]. The medial OFC has strong connections with limbic regions, receives reciprocal connections from the DLPFC, and projects to the hypothalamus and periaquaductal gray [6,9,10,12]. It is thought to be involved in emotional regulation of visceral function [4], and in the monitoring, learning and memory of the reward value of reinforcers [7]. The OFC is also impacted by the experience of stress. Structural changes in both the lateral and medial OFC networks have been associated with prior experience of early life trauma or chronic stress [13-15].

Stressful experiences may not only be linked to structural changes in the OFC, but have also been shown to increase risk for, and contribute to the onset of, schizophrenia and bipolar disorder. A number of retrospective and prospective epidemiological studies have demonstrated that early life stress increases the risk of psychosis later in life [16-20], while stress later in life can impact the course of psychotic illness [21,22]. In many individuals with schizophrenia or bipolar disorder, dysregulation of the hypothalamic-pituitary-adrenal (HPA) axis, the primary hormonal stress response pathway, is also observed [23-25]. Prior studies have not directly determined whether molecular abnormalities within the HPA axis stress signalling pathway are present in the OFC in schizophrenia or bipolar disorder.

Although molecular aspects of stress signalling have not been investigated in the OFC in psychiatric illness, other structural, functional and molecular changes within the medial and lateral OFC networks have been reported in bipolar disorder and schizophrenia. Structural OFC changes, such as reduced gray matter density and reduced white matter fractional anisotropy, have been identified in individuals with bipolar disorder relative to healthy controls [26,27]. Decreased functional activation of the lateral OFC network, and altered functional connectivity of the medial OFC network with the amygdala, are observed in bipolar disorder and schizophrenia during emotion processing and regulation tasks [28-30]. Decreased metabolic activity in the OFC networks of drug-free schizophrenia patients has also been reported [5]. At a molecular level, decreased glutamic acid decarboxylase 67 (GAD 67) expression has been reported in the lateral OFC in schizophrenia and bipolar disorder [31], while increased dopamine receptor (D3 and D4) mRNA, serotonin receptor 1A binding, N-methyl-D-aspartate receptor binding and metabotropic glutamate receptor protein expression have been identified throughout the OFC in schizophrenia [32-36]. Furthermore, OFC neurons have been identified as the targets for a number of antipsychotic drugs [37]. These findings suggest a role for the lateral OFC network in the pathogenesis of both bipolar disorder and schizophrenia, and highlight the potential relevance of targeting OFC neurons when treating psychotic illness.

Despite the relevance of the OFC in bipolar disorder and schizophrenia, and the potential role of stress in the pathogenesis of these disorders, it is not known whether molecular abnormalities of the stress response pathway exist in the OFC. Recent evidence of such abnormalities in other brain regions, involving the primary stress receptor, the glucocorticoid receptor (GR), has emerged in schizophrenia and bipolar disorder. A network of regions, including the hippocampus, amygdala and temporal cortex, display decreased total GR mRNA expression in both schizophrenia and bipolar disorder [38-40]. In contrast, total GR mRNA expression is decreased in the entorhinal cortex in bipolar disorder but not schizophrenia, and is decreased in the DLPFC in schizophrenia but is not significantly changed in bipolar disorder [38,41]. Levels of GRα protein have only been examined in the DLPFC, in which increases of a functional truncated GRα isoform, putative GRα-D1, are observed in both schizophrenia and bipolar disorder [40]. It is not known whether GR mRNA and protein abnormalities occur in the OFC in psychotic illness, and to what extent GR expression patterns in the OFC mirror those observed in the DLPFC and/or in other brain regions.

Based on evidence of structural and functional abnormalities of the lateral OFC network in psychiatric illness, its functional relevance to psychiatric illness as an integrator of sensory information, and its possible sensitivity to stress, we hypothesised that GR mRNA and protein dysregulation would be evident in the lateral OFC, as in the DLPFC, in schizophrenia and bipolar disorder. Therefore, in this study we explored GR expression in lateral OFC in both schizophrenia and bipolar disorder, using a cohort of 104 post-mortem samples. We sought to 1) determine whether expression levels of specific GR exon 1 mRNA transcript variants are altered in the lateral OFC in schizophrenia and bipolar disorder cases relative to controls, 2) quantify expression of GRα protein isoforms in the lateral OFC in schizophrenia and bipolar disorder compared to controls, and 3) determine if selected human GR gene (NR3C1) polymorphisms relate to GR mRNA or protein expression in the lateral OFC.

## Methods

### Tissue collection

These studies were carried out in accordance with the declaration of Helsinki, after approval by the Human Research Ethics Committee at the University of NSW (#HREC07261). Written informed consent for use of tissues in the study was obtained from next of kin. Brain samples from the Stanley Medical Research Institute (SMRI) Array Cohort were collected by pathologists in the Office of the Medical Examiner in several states [42]. The selection process, clinical information, diagnoses of patients and processing of tissues have been described previously [42]. DSM-IV diagnoses were made independently by two senior psychiatrists based on medical records and, when necessary, telephone interviews with family members. Exclusion criteria included anyone over age 70, anyone with a history of seizures or other neurologic disorders that might affect brain pathology, and anyone with evidence of such conditions on neuropathologic examination. Diagnostic groups did not differ significantly according to age, RIN, PMI, hemisphere or brain weight, and were balanced for gender, race, and hemisphere. Brain pH was significantly lower in the schizophrenia and bipolar disorder groups than in the control group (both *p* < 0.05). There were a significantly greater number of female cases in the bipolar disorder group than in the schizophrenia or control groups (*p* < 0.05). The SMRI supplied total RNA, genomic DNA and crude protein homogenate from 35 schizophrenia cases, 34 bipolar disorder cases and 35 control individuals (Table 1). The region of the lateral OFC sampled was between the branches of the orbital sulcus, in BA11L as defined by Öngur and Price [43].

**Table 1:** Demographic details of cases used in this study

	**Control group**	**Bipolar disorder group**	**Schizophrenia group**
	**(n = 35)**	**(n = 34)**	**(n = 35)**
Diagnostic subtype	-	BP1 = 27, BP2 = 4, BPNOS = 2, schizoaffective = 1	SCZ(disorganised) = 1, SCZ(paranoid) = 8, SCZ(undifferentiated) = 26
Age (years)	44.2 (31–60)	45.4 (19–64)	42.6 (19–59)
Gender	9 F, 26 M	18 F, 16 M	9 F, 26 M
Hemisphere	16 L, 19R	19 L, 15R	17 L, 18R
pH	6.61 +/− 0.27	6.43 +/− 0.30	6.48 +/− 0.24
PMI (hours)	29.4 +/− 12.9	37.9 +/− 18.6	31.4 +/− 15.5
RIN	7.23 +/− 0.87	7.34 +/− 0.88	7.36 +/− 0.61
Manner of death	natural = 35	natural = 19, suicide = 15	natural = 28, suicide = 7
Age of onset (years)	-	25.3 +/− 9.2	21.3 +/− 6.1
Duration of illness (years)	-	20.2 +/− 9.6	21.3 +/− 10.2
Lifetime antipsychotics (fluphenazine equivalents, mg)	-	10212 +/−22871	85004 +/− 100335
Antidepressant use	yes = 0, no = 35	yes = 19, no = 15	yes = 9, no = 26
Type of antidepressant*	-	SSRI = 9 (fluoxetine = 5) SNRI = 4, SARI = 5, TCA = 6, other = 1	SSRI = 4 (fluoxetine = 2), SNRI = 0, SARI = 2, TCA = 2, other = 2
Smoking around time of death	yes = 9, no = 9, unknown = 17	yes = 15, no = 6, unknown = 13	yes = 23, no = 4, unknown = 8

Demographic details of controls, bipolar disorder cases and schizophrenia cases used in this study. *Note- some individuals took multiple antidepressant medications. Abbreviations: BP1 = bipolar disorder type I, BP2 = bipolar disorder type II, BPNOS = bipolar disorder not otherwise specified, SCZ = schizophrenia, M = male, F = female, L = left, R = right, PMI = post-mortem interval, RIN = RNA integrity number, SSRI = selective serotonin reuptake inhibitor, SNRI = serotonin–norepinephrine reuptake inhibitor, SARI = serotonin antagonist and reuptake inhibitor, TCA = tricyclic antidepressant. Data quoted are mean (range) +/− standard deviation.

### Endpoint PCR analysis

Endpoint PCR was performed to amplify GR exon 1 mRNA transcript variants in cDNA from schizophrenia, bipolar disorder and control OFC tissue (pooled from all cohort samples) and from universal human cDNA from normal human tissues (Biotaq, Gaithersburg, MD). Primer sequences were as follows: GR-A forward primer ATCACTTTCACTTCTGCTGG, reverse primer CAGTGGATGCTGAACTCTTGG, GR-1B forward primer GCCGGCACGCGACTCC reverse primer CAGTGGATGCTGAACTCTTGG, GR-1C_1-3_ (detecting all GR-1C variants and henceforth called simply GR-1C) forward primer GCTCCTCTGCCAGAGTTGAT reverse primer CAGTGGATGCTGAACTCTTGG, GR-1D forward primer ACAACCTTTCCCAGAGTC reverse primer CAGTGGATGCTGAACTCTTGG, GR-1E forward primer CGTGCAACTTCCTTCGAGT reverse primer CAGTGGATGCTGAACTCTTGG, GR-1F forward primer GTAGCGAGAAAAGAAACTGG reverse primer CAGTGGATGCTGAACTCTTGG, GR-1H forward primer CTGACAGCCCGCAACTTGGA reverse primer CAGTGGATGCTGAACTCTTGG. Each reaction contained forward and reverse primers (0.2 mM), cDNA (approximately 4.5 ng/μl), dNTPs (0.2 mM), MgCl_2_ (2 - 4 mM) and RedHot DNA polymerase (0.5 U; Thermo Scientific, Waltham, MA) in 1x reaction buffer. The reaction mix including cDNA was incubated at 94°C (3 min), followed by 40 cycles of 94°C (30 s), 53 - 62°C (30 s, or 90 s for GR-1A) and 72°C (30 s), then 72°C (10 min) and 4°C overnight. Products were run on a 1% agarose gel alongside a 100 bp ladder (Fermentas, Waltham, MA), and visualised on the Chemidoc XRS Molecular Imager (Bio-Rad, Hercules, CA).

### Quantitative real-time PCR (qPCR) analysis

Total RNA was extracted by the SMRI from lateral OFC tissue of 34 schizophrenia cases, 31 bipolar cases and 34 controls using Trizol Reagent (Invitrogen, Carlsbad, CA). cDNA was then synthesised from total RNA using the Superscript First-Strand Synthesis Kit (Invitrogen, Carlsbad, CA), and qPCR analysis conducted as previously reported [44]. Pre-designed TaqMan gene expression assays (Applied Biosystems, Foster City, CA) targeting pan GR (cat. # Hs00230818_m1), GR-1B (Hs01005211_m1) and GR-1C (Hs01010775_m1) were used. Custom Taqman primer/probes were also designed to target the exon 1–2 boundary of GR-1F (forward primer, CTCGGTGGCCCTCTTAACG; reverse primer, CAGGAGTTAATGATTCTTTGGAGTCCAT; probe, CAGAGAGACCAGTTGATATT) and GR-1H (forward primer, GCGTGTCGGAGAGAGAACT; reverse primer, GGGTTTTCTTCTCTACCAGGAGTTA; probe, TCCATCAGTGAATATCAACTGTT). Four ‘housekeeper’ genes: β-actin (ACTB; Hs99999903_m1), beta-2-microglobulin (B2M; Hs99999907_m1), TATA-binding protein (TBP; Hs00427620_m1) and ubiquitin C (UBC; Hs00824723_m1) were assayed. Serial dilutions of cDNA, pooled from all cohort samples, were included on every qPCR plate for quantification of sample expression by the relative standard curve method. For qPCR gene expression analysis, reactions were performed in triplicate. Normalisation to the geometric mean of four housekeeper genes was then performed, and population outliers excluded if their normalised expression values were greater than 2 standard deviations from the group mean. For each analysis, between 32–34 control individuals, 30–31 individuals with bipolar disorder and 31–33 individuals with schizophrenia were retained after outlier removal. To estimate the relative abundances of each GR mRNA variant within each individual, relative amounts of each transcript were calculated by the 2^-ΔΔCt^ method [45], using the ACTB housekeeper as internal control gene and pan GR mRNA as the calibrator.

### Western blotting

Western blotting was conducted as previously described [40,46], using crude protein homogenates supplied by the SMRI from the lateral OFC of 35 schizophrenia cases, 34 bipolar cases and 35 controls. The P-20 anti-GRα primary antibody (sc-1002X, Santa Cruz Biotechnology, Santa Cruz, CA) was used for detection of GRα in this study. Antibody specificity has been previously demonstrated, with amelioration of GRα immunoreactivity by pre-incubation of this same P-20 antibody batch with blocking peptide [40]. Seven micrograms of protein homogenate was heated (95°C, 5 min), run on 10% bis-tris polyacrylamide gels (Bio-Rad) and transferred onto nitrocellulose membranes (Bio-Rad) at 100 V for 30 min. Blots were probed with the P-20 anti-GRα primary antibody (1:2000 dilution in 5% skim milk), followed by goat anti-rabbit secondary (1:2000; Millipore, Billerica, MA). After stripping (stripping buffer: 25 mM glycine, 1.5% sodium dodecyl sulfate, pH 2.0), blots were incubated with anti-β-actin primary antibody (1:10000; MAB1501, Millipore), followed by goat anti-mouse secondary (1:5000; Millipore). Blots were exposed to autoradiographic film (Amersham, Bucks, UK) and quantified using Quantity One analysis software (Bio-Rad). Duplicate samples were run in separate experimental runs, with the same batch of each antibody used for all runs. Within each run the total intensity of each immunoreactive band was normalised to an internal control (pooled sample from entire cohort) loaded onto the same gel, and to the β-actin band detected in the same lane. The average β-actin across the two experimental runs did not significantly differ between diagnostic groups. Population outliers in each diagnostic group were excluded if the sample normalised quantity value was greater than 2 standard deviations from the group mean. The geometric mean of both runs was then calculated, expressed as a percentage of the control mean for each band. For each analysis, between 34–35 control cases, 33–34 bipolar disorder cases and 34–35 schizophrenia cases were retained after outlier removal.

### Genotyping

Eleven putative functional SNPs in the NR3C1 gene were chosen for genotyping (Table 2) using genomic DNA from 34 schizophrenia cases, 30 bipolar cases and 31 controls. Methods employed for DNA extraction and genotyping were described when this genotype data were first published [41]. Genomic DNA was extracted by the SMRI with the Promega Wizard genomic kit (Promega, Madison, WI). Briefly, genotyping was performed with 20 ng of genomic DNA, in a multiplex assay using a Sequenom MassArray, Autoflex Spectrometer and iPLEX GOLD chemistry. The pass rate for genotyped samples was 99.0%. PLINK (version 1.06, http://pngu.mgh.harvard.edu/purcell/plink) [47] was used for Hardy-Weinberg equilibrium testing.

**Table 2:** Details of NR3C1 (GR) SNPs analysed in this study

**dbSNP rs #**	**location (UCSC build Hg19, Feb 2009**	**location, description, (common name)**	**poly-morphism (major/minor allele)**	**MAF**	**HWE *p-* value**
rs10052957	142786701	5' UTR; 2656 bases upstream of exon 1B (Tth111l)	C/T	0.320	1
rs72801094	142785905	5' UTR; 1860 bases upstream of exon 1B	A/G	0.046	0.177
rs5871845	142783949	5' UTR; exon 1B	-/C	0.052	1
rs10482614	142782402	5' UTR; between exon 1 C and exon 1 H	G/A	0.134	0.012
rs10482616	142781567	5' UTR; between exon 1 H and exon 2	G/A	0.113	0.342
rs4634384	142780697	5' UTR; between exon 1 H and exon 2	G/A	0.490	0.687
rs6190	142780337	exon 2; non-synonymous (R23K)	G/A	0.021	1
rs1800445	142779311	exon; non-synonymous (N363S)	A/G	0	-
rs41423247	142778575	intron; between exon 2 and exon 3, 645 bases downstream of exon 2 (Bcl1)	C/G	0.345	0.825
rs6196	142661490	exon 9α; synonymous (N766N)	T/C	0.139	0.017
rs6198	142657621	3' UTR; inside exon 9β (A369G)	A/G	0.170	0.065

Details of genotyped SNPs. Abbreviations: UCSC = University of California Santa Cruz, MAF = minor allele frequency, HWE = Hardy-Weinberg Equilibrium, UTR = untranslated region.

### Statistical analysis

All data were approximately normally distributed (skewness between −1.0 and 1.0). Pearson correlation analyses were conducted with normalised mRNA or protein levels and age, pH, PMI and RIN values. In addition, within schizophrenia and bipolar disorder groups, correlation analyses were performed with normalised mRNA or protein levels and age-of-onset, duration-of-illness and fluphenazine-equivalent antipsychotic drug measures. If significant correlations with demographic variables were observed, analysis of covariance (ANCOVA) with Fisher’s LSD post-hoc analysis was used to determine group differences according to diagnosis, gender, hemisphere and smoking. To determine effects of manner of death and antidepressants, ANCOVA and LSD post-hoc tests were performed after the schizophrenia and bipolar disorder groups were sub-divided according to suicide status (positive/negative) or history of antidepressant use (positive/negative). Analysis of variance (ANOVA) was used if no correlations were seen. Main effects ANOVAs, with diagnosis and genotype as independent factors, were used to identify the effects of genotype on mRNA and protein expression, and also identify any genotype-diagnosis interactions.

## Results

### GR mRNA expression in the OFC in schizophrenia and bipolar disorder

**Figure 1:**
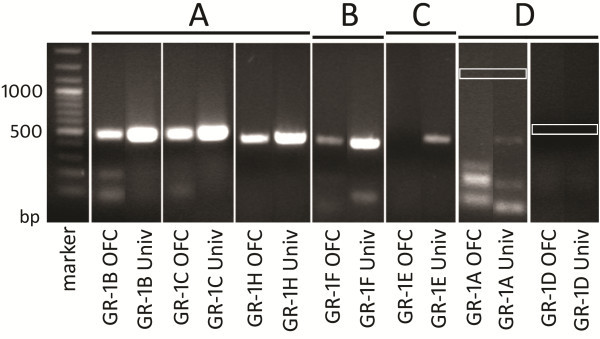
**Endpoint PCR detection of GR exon 1 mRNA transcript variants in the OFC. A**) Strong amplification of the GR-1B, GR-1C and GR-1H variants was evident in OFC cDNA and in universal human cDNA. **B**) Weaker amplification of the GR-1F variant was observed in the OFC, while strong GR-1F amplification was seen in universal human cDNA. **C**) No amplification of GR-1E was seen in OFC cDNA, while weak amplification was seen in universal human cDNA, **D**) No amplification of GR-1A or GR-1D in OFC cDNA or universal human cDNA was detected. Boxes indicate expected amplicon sizes. Abbreviations: Univ- universal human cDNA, bp- base pairs.

We determined by endpoint PCR that the GR-1B, GR-1C, GR-1F and GR-1H mRNA transcript variants are expressed in the OFC (Figure 1). The GR-1B, GR-1C, GR-1F and GR-1H mRNA transcripts were abundant in universal human cDNA, which was used as a positive control. GR-1E was not detected in OFC tissue but was present in universal cDNA. GR-1A_1-3_ and GR-1D were not detected in OFC tissue or universal cDNA (Figure 1). Expression levels of the GR-1B, GR-1C, GR-1F and GR-1H mRNA transcripts were then quantified by qPCR.

**Figure 2:**
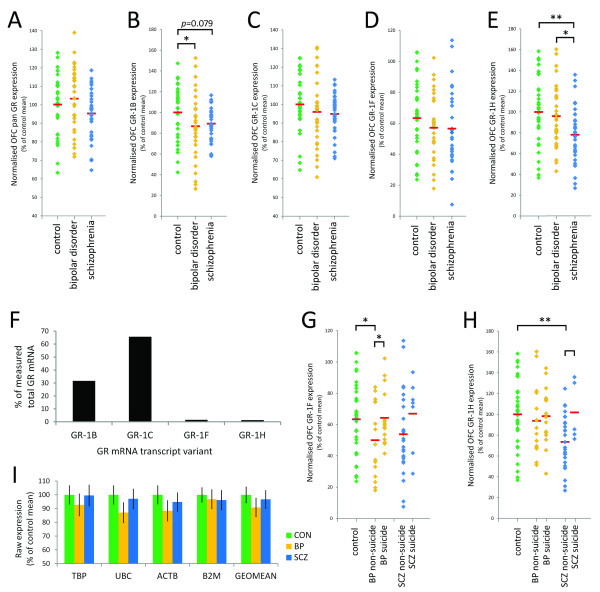
**Expression of GR mRNA in the OFC in controls, bipolar disorder cases and schizophrenia cases.** Expression of GR mRNA in the OFC of controls (green diamonds), bipolar disorder cases (orange diamonds) and schizophrenia cases (blue diamonds). **A**) A trend towards an effect of diagnosis on pan GR in the OFC was observed (ANCOVA, p = 0.07), but no significant differences were seen in planned post-hoc tests comparing controls to schizophrenia or to bipolar disorder cases; **B**) There were subtle diagnostic differences in GR-1B mRNA expression in the OFC, with a 14.3% decrease in GR-1B expression in bipolar disorder cases relative to controls (p < 0.05) and a non-significant decrease in schizophrenia cases relative to controls (10.8%, p = 0.079); **C** and **D**) No significant diagnostic differences in GR-1C or GR-1F mRNA expression in the OFC were observed; **E**) GR-1H mRNA expression in the OFC was significantly decreased in schizophrenia cases relative to controls (22.0% decrease, p < 0.005) and in schizophrenia cases relative to bipolar disorder cases (19.5% decrease, p < 0.05); **F**) GR-1B, GR-1C, GR-1F and GR-1H mRNA transcript variants represented approximately 31.6%, 65.6%, 1.6% and 1.2% of total measured GR mRNA in the OFC respectively; **G**) Suicide-negative bipolar disorder (BP non-suicide) cases displayed 24.6% lower GR-1F mRNA than controls (p < 0.05), and 25.6% lower than suicide-positive bipolar disorder (BP suicide) cases (p < 0.05); **H**) Suicide-negative schizophrenia (SCZ non-suicide) cases displayed 26.5% lower GR-1H mRNA than controls (p < 0.001), and 27.9% lower than suicide-positive schizophrenia (SCZ suicide) cases (p < 0.05). **I**) There were no diagnostic differences in raw mRNA expression levels of housekeeping genes TBP, UBC, ACTB or B2M individually, nor the geomean of all four. Abbreviations: BP- bipolar disorder, SCZ- schizophrenia, CON- control. * p < 0.05, ** p < 0.005.

For analysis of GR mRNA levels, in order to control for variation in input material between samples, data were normalised to the geomean of raw expression values for TBP, UBC, ACTB and B2M mRNAs. No housekeeper individually, nor the geomean of all four, varied significantly between diagnostic groups (Figure 2I).

Next, we determined whether OFC GR mRNA transcript expression was influenced by brain cohort demographic variables. RIN was significantly correlated with levels of all transcripts (all r ≤ 0.27, *p* < 0.05), while brain pH was significantly correlated with pan GR, GR-1B and GR-1F mRNA transcript levels (all r ≤ 0.23, *p* < 0.05). Brain weight, age and PMI did not correlate with any GR mRNA measures.

Significant diagnostic group differences in expression of GR mRNA transcript variants were identified in the lateral OFC. For normalised pan GR mRNA, diagnostic group differences in expression approached significance [ANCOVA F(2, 89) = 2.74, *p* = 0.07, co-varying for RIN and brain pH; Figure 2A]. In planned post-hoc comparisons comparing controls to schizophrenia or bipolar disorder cases, no significant differences were identified (both *p* > 0.17). However, there were diagnostic differences in GR-1B and GR-1H mRNA transcript variant levels in the lateral OFC. The effect of diagnosis on GR-1B mRNA expression did not reach significance [ANCOVA F(2, 87) = 2.17, *p* = 0.12, co-varying for RIN and brain pH]. However, in planned post-hoc comparisons, a significant 14.3% decrease in GR-1B expression in bipolar disorder cases relative to controls was observed (*p* < 0.05; Figure 2B). A trend towards a reduction in GR-1B mRNA expression was observed in schizophrenia cases relative to controls (10.8% decrease, *p* = 0.079). A trend towards decreased GR-1F mRNA expression in schizophrenia and bipolar disorder cases was seen [ANCOVA F(2, 90) = 2.56, *p* = 0.08, co-varying for RIN and brain pH; Figure 2D], but no significant group differences were observed by post-hoc test. For the GR-1H mRNA transcript variant, significant group differences in expression according to diagnosis were seen [ANCOVA F(2, 90) = 5.72, *p* < 0.005, co-varying for RIN]. GR-1H mRNA expression was decreased in schizophrenia cases relative to controls (22.0%, *p* < 0.005) and in schizophrenia cases relative to bipolar disorder cases (19.5%, *p* < 0.05; Figure 2E). No effect of diagnosis on GR-1C mRNA expression was observed [ANCOVA F(2, 91) = 1.22, *p* = 0.30, co-varying for RIN; Figure 2C].

The relative abundances of each GR mRNA transcript variant, relative to total measured GR mRNA, were estimated within each individual. On average, the GR-1B, GR-1C, GR-1F and GR-1H mRNA transcript variants represented approximately 31.6%, 65.6%, 1.6% and 1.2% of total measured GR mRNA in the OFC respectively (Figure 2F).

### Effects of suicide and other cohort demographic variables on GR mRNA expression

A significant effect of suicide status on GR-1H mRNA in the lateral OFC was observed [ANCOVA F(4, 88) = 3.70, co-varying for RIN, *p* < 0.01; Figure 2H]. Suicide-negative schizophrenia cases displayed 26.5% lower GR-1H mRNA than controls (*p* < 0.001), and 27.9% lower than suicide-positive schizophrenia cases (*p <* 0.05). A trend towards an effect of suicide on GR-1F mRNA was also seen [ANCOVA F(4, 88) = 1.92, co-varying for RIN, *p =* 0.11; Figure 2G]. By post-hoc test, suicide-negative bipolar disorder cases displayed 24.6% lower GR-1F mRNA than controls (*p* < 0.05), and 25.6% lower than suicide-positive bipolar cases (*p* < 0.05). No significant differences in pan GR, GR-1B or GR-1C mRNA expression between suicide-positive and suicide-negative schizophrenia or bipolar disorder cases were detected. When diagnostic groups were subdivided according to presence/absence of antidepressant use, no significant group differences in pan GR, GR-1B, GR-1C, GR-1F or GR-1H mRNA expression in the lateral OFC were observed (all *p* > 0.05). When the schizophrenia and bipolar disorder groups were combined and divided according to fluoxetine use, no group differences in pan GR, GR-1B, GR-1F or GR-1H GR mRNA expression of individuals on fluoxetine (n = 7), compared with individuals not on fluoxetine (n = 58), were observed. However a significant 16.3% increase in GR-1C mRNA expression in individuals on fluoxetine (n = 7) relative to with individuals not on fluoxetine was seen [ANCOVA F(1, 59) = 6.72, *p* < 0.05]. No differences in pan GR, GR-1B, GR-1C, GR-1F or GR-1H mRNA expression according to gender, hemisphere or smoking status were observed (all *p* > 0.05). In bipolar disorder cases, significant negative correlations were seen with age of onset for pan GR and GR-1B mRNA levels (r = −0.392, *p* < 0.05 and r = −0.387, *p* < 0.05 respectively), and with time in hospital for GR-1C mRNA levels (r = −0.363, *p* < 0.05). No significant correlations of lifetime antipsychotic exposure with pan GR, GR-1B, GR-1C, GR-1F or GR-1H mRNA levels in bipolar disorder cases were seen (all *p* > 0.05). Within the schizophrenia group, GR-1B mRNA levels were significantly negatively correlated with duration of illness (r = −0.42, *p* < 0.05), but not with age of onset, time in hospital or level of lifetime antipsychotic exposure. No significant correlation of pan GR, GR-1C, GR-1F or GR-1H mRNA levels with duration of illness, age of onset, time in hospital or level of lifetime antipsychotic exposure in schizophrenia cases were seen. No significant group differences were observed in pan GR, GR-1B, GR-1C, GR-1F and GR-1H mRNA levels when samples were grouped according to lifetime illicit drug or alcohol use.

### GRα protein isoforms in the OFC in schizophrenia and bipolar disorder

**Figure 3:**
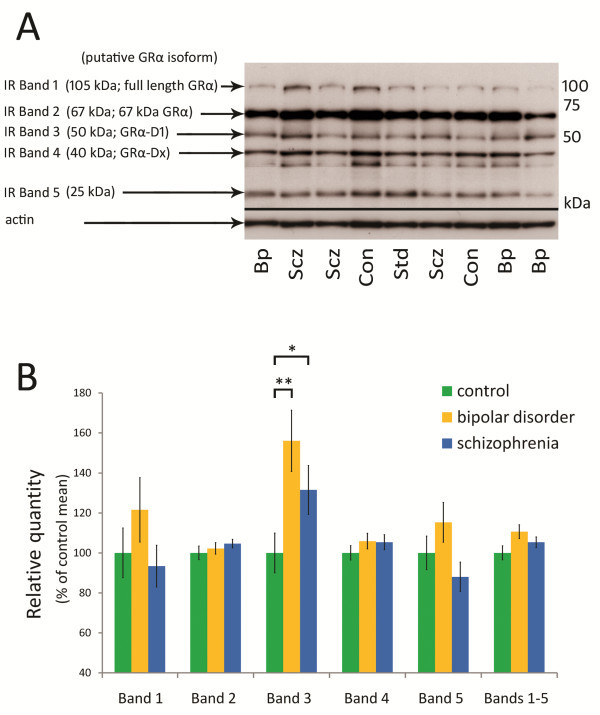
**Quantification of GRα protein in the OFC in controls, bipolar disorder cases and schizophrenia cases. A**) Representative western blot of lateral OFC protein homogenate, revealing immunoreactive (IR) bands 1–5, approximately 105, 67, 50, 40 and 25 kDa in size. IR bands 1, 2, 3 and 4 are likely to represent full-length GRα, 67 kDa GRα, GRα-D1 and GRα-Dx respectively. **B**) Intensities of GRα IR bands in western blotting of lateral OFC samples. There were significant differences in intensities of IR band 3 [GRα-D1; ANOVA F(2, 97) = 4.15, *p* < 0.05] between schizophrenia cases, bipolar disorder cases and controls. A significant 56.1% increase in IR band 3 intensity of bipolar disorder cases relative to controls (*p* < 0.005), and a significant 31.5% increase in IR band 3 intensity of schizophrenia cases relative to controls (*p <* 0.05) were observed. Error bars represent SEM. Abbreviations: Bp- bipolar disorder, Scz- schizophrenia, Con- control.* p < 0.05, ** p < 0.005.

We tested if GRα protein isoform abnormalities exist in the lateral OFC by quantifying GRα protein expression in the lateral OFC using western blotting. Using this technique previously, in combination with cloning and in vitro expression, we established that immunoreactive (IR) band 1 is likely to represent the full-length GRα, and IR band 2 represents an uncharacterised 67 kDa isoform [46]. Of the smaller isoforms, IR band 3 putatively represents the truncated GRα-D1 isoform, IR band 4 represents another GRα-D isoform (GRα-Dx) and IR band 5 represents an uncharacterised 25 kDa isoform (Figure 3A).

In the lateral OFC, GRα protein measures were not significantly correlated with age, brain pH, PMI or brain weight, with the exception of IR band 3 intensity, which was positively correlated with PMI (r = 0.21, *p* < 0.05), accounting for a small amount of variance. No significant differences between schizophrenia, bipolar disorder and control cases were observed in intensities of IR bands 1, 2, 4 and 5 or the sum of IR bands 1–5 (Figure 3B). However, as in the DLPFC, there were significant diagnostic group differences in IR band 3 (GRα-D1) intensity [ANCOVA F(2, 97) = 4.15, *p* < 0.05]. IR band 3 intensity was increased in bipolar disorder cases relative to controls (56.1%, *p* < 0.005, one-tailed *t*-test), and in schizophrenia cases relative to controls (31.5% *p* < 0.05, one-tailed *t*-test). No correlations of OFC GRα protein measures with age of onset, duration of illness or level of lifetime antipsychotic exposure within schizophrenia and bipolar disorder cases were observed.

GR mRNA expression levels in the lateral OFC did not correlate significantly with abundance of GRα protein isoforms, with the exception of pan GR mRNA expression, which correlated positively with GRα IR band 5 abundance (r = 0.23, *p* < 0.05).

### Relationships between NR3C1 gene polymorphisms, GR mRNA expression and GRα protein isoform abundance in the OFC

The relationship between NR3C1 genotype and GR mRNA expression was explored in the lateral OFC using genotype data for 11 single nucleotide polymorphisms (SNPs, Table 2). When analysed by main effects ANOVAs, there were no significant relationships between NR3C1 genotype (at any of the genotyped SNPs) and expression of pan GR, GR-1B, GR-1C or GR-1H mRNA transcript variants in the lateral OFC. For the rs10052957 (Tth111l) SNP, a significant, dose-dependent relationship between genotype and GR-1B mRNA expression was previously seen in the DLPFC. For this rs10052957 SNP, there were no main effects of genotype on pan GR [F(2, 80) = 0.96, *p* = 0.39], GR-1B [F(2, 78) = 0.68, *p* = 0.51], GR-1C [F(2, 82) = 0.44, *p* = 0.65] or GR-1H [F(2, 80) = 0.048, *p* = 0.95].

Possible effects of NR3C1 genotype on GRα protein measures were also explored in the lateral OFC. There were no significant main effects of NR3C1 genotype on intensities of IR band 1, 2, 3, 4, 5 or total IR bands 1–5 in the OFC. Previously, we identified an NR3C1 SNP, rs41423247 (Bcl1), which had a possible effect on GRα protein expression in the DLPFC [41]. In the lateral OFC, this SNP was not associated with differences in abundance of IR band 1 [F(2, 86) = 1.54, *p* = 0.22], IR band 2 [F(2, 85) = 0.69, *p* = 0.50], IR band 3 [F(2, 85) = 0.69, *p* = 0.50], IR band 4 [F(2, 86) = 0.61, *p* = 0.61], IR band 5 [F(2, 85) = 0.73, *p* = 0.49] or total IR bands 1–5 [F(2, 85) = 0.43, *p* = 0.65].

## Discussion

In this study, we identified abnormalities of GR mRNA and protein expression in bipolar disorder and schizophrenia in the lateral OFC. These abnormalities particularly implicated the GR-1B mRNA transcript variant in bipolar disorder, the GR-1H mRNA transcript variant in schizophrenia, and the GRα-D1 protein isoform in both bipolar disorder and schizophrenia. Interestingly, the transcript-specific patterns of GR mRNA dysregulation within the lateral OFC in bipolar disorder and schizophrenia differed from the more generalised GR mRNA dysregulation identified in the DLPFC in these disorders [41].

In bipolar disorder, decreased expression of the GR-1B mRNA transcript variant was identified in the lateral OFC, while no changes were observed in levels of pan GR, GR-1C or GR-1H mRNA transcripts. This pattern contrasts with the dysregulation seen in the DLPFC, which was characterised by decreased GR-1C and GR-1H mRNA transcripts in bipolar disorder, a subtle decrease in pan GR mRNA, and no change in GR-1B mRNA [41]. These findings suggest that distinct dysregulation of GR mRNA expression occurs in the lateral OFC and DLPFC in bipolar disorder, and implicate the transcriptional regulatory mechanisms governing GR-1B mRNA expression in the lateral OFC in bipolar disorder. GR-1B mRNA transcript expression is driven by a unique upstream promoter region [48], and can be influenced by sequence variation in the NR3C1 promoter region in the human DLPFC [41]. Furthermore, GR mRNA transcript variants, including GR-1B, are likely to be also regulated by tissue-specific transcription factors, which mediate tissue-specific GR action [49]. It is possible that these regulatory mechanisms, and/or others yet to be defined, are involved in dysregulation of GR-1B mRNA in bipolar disorder. GR promoter methylation may also play a role, since GR promoter hyper-methylation has been associated with decreased GR-1B mRNA expression in the hippocampus of child abuse sufferers [50]. Greater variation in GR-1B mRNA levels was observed in bipolar disorder cases than schizophrenia cases in the lateral OFC. This was not a gender effect, since males and females displayed equivalent variability in GR-1B mRNA expression in bipolar disorder. However, it may have been due in part to the influence of age of illness onset on GR-1B expression in bipolar disorder, since more cases with later age of onset are present in the bipolar disorder group than the schizophrenia group, and decreased GR-1B mRNA correlated with later age of onset. However, the effect of this illness parameter on GR-1B expression is difficult to interpret, since earlier age of onset has been linked to increased illness severity [51], but is associated with higher GR-1B mRNA levels among bipolar disorder cases, more reminiscent of normal controls. Although GR-1B expression (which represents approximately 32% of total measured GR mRNA) was decreased in bipolar disorder, there was no difference in pan GR mRNA levels between bipolar disorder cases and controls. This may arise because other GR mRNA transcripts including GR-1C (which represents approximately 66% of total measured GR mRNA) may have diluted this diagnostic effect, despite themselves being unchanged in bipolar disorder.

In schizophrenia, a significant decrease in levels of the GR-1H mRNA transcript variant, along with a trend towards a decrease in GR-1B mRNA expression, were observed in the lateral OFC. As observed for bipolar disorder, these mRNA abnormalities in schizophrenia were more circumscribed in the lateral OFC than in the DLPFC, where decreases in pan GR mRNA and all transcript variants (GR-1B, GR-1C and GR-1H) were observed [41]. The GR-1H transcript includes exon 1H, the GR alternative first exon which is located most proximal to exon 2. The regulation of GR-1H expression by transcription factors in its promoter region, and the function of GR-1H, have not been characterised. Furthermore, GR-1H mRNA represents only a small fraction (approximately 1.2%) of total measured GR mRNA in this study, as in other studies [52]. As a result, the selective GR-1H mRNA deficits which we observe may have limited impact on GR signalling in the lateral OFC in schizophrenia.

The effects of antidepressant use in general, and fluoxetine use in particular, were explored in this study, since previous work has showed selective effects of fluoxetine on total GR and GR-1F mRNA expression in rodent hippocampus [53]. In our study, GR-1C mRNA expression was increased in fluoxetine users relative to non-users. The direction of this change is consistent with the previous study. However, unlike previously reported, we observed no effects of fluoxetine on GR-1F mRNA expression in the OFC, suggesting that antidepressant effects may vary between species and/or brain regions.

The primary GRα protein abnormality that we observed was increased abundance of a truncated GRα isoform, putative GRα-D1, in both bipolar disorder and schizophrenia in the OFC. This same increase in GRα-D1, of a similar magnitude, was seen in bipolar disorder and schizophrenia cases in the DLPFC [40]. In vitro experiments have revealed that the abundance of the GRα-D1 isoform is determined not only by mRNA transcript levels, but also by post-transcriptional mechanisms [46,54]. Consistent with these findings, over-expression of the GRα-D1 isoform was observed in both the DLPFC and lateral OFC in bipolar disorder and schizophrenia, despite divergent patterns of GR mRNA dysregulation in both regions. The absence of consistent correlations between GR mRNA and GRα protein measures in this study also suggests post-transcriptional regulation of GRα protein abundance. GRα-D1 has been previously shown to function as a transcription factor at glucocorticoid response elements [40], and to activate and repress the transcription of numerous target genes [54]. As a result, upregulation of GRα-D1 has the potential to influence diverse aspects of cellular function. Since the OFC is an integral component of the brain’s reward circuitry [2], and is involved in integrating sensory information [4,6], it is possible that abnormal GR signalling may impact these cognitive functions, particularly during the experience of stress.

In both psychotic illnesses, a lesser involvement of the lateral OFC than the DLPFC in GR mRNA deficits was seen. One possible reason for this observation may relate to the experience of stress. It is plausible that more widespread GR mRNA dysregulation in the DLPFC than the lateral OFC arises due to chronic illness-induced stress in both illnesses. Stress has been shown to down-regulate GR mRNA expression [55-58]. The DLPFC may be more sensitive to this effect, having stronger connections than the lateral OFC (BA11L) with other stress-sensitive regions which are involved in regulating HPA axis activity, such as the hippocampus and hypothalamus [3,4,6,9,10]. To explore this possibility we examined the relationship between GR mRNA expression and suicide, which may be associated with stressful experiences prior to death. We observed an influence of suicide on expression of multiple GR mRNA transcript variants in the DLPFC [41], but this influence was limited to the GR-1F and GR-1H mRNA transcripts in the lateral OFC, suggesting that the lateral OFC may be less sensitive than the DLPFC to the (stressful) effects of suicide. In all cases, GR mRNA or GRα protein changes in individuals with suicide were in the opposite direction to diagnostic differences, and therefore were not driving diagnostic group differences. Taking into account the divergent GR mRNA and protein findings in this study, and in previous work in the DLPFC [40,41], it is likely that multiple processes are involved in GR dysregulation in the frontal cortex in psychotic illness, potentially representing a mix of primary, consequential and/or compensatory changes. Some of these changes may be anatomically specific, whereas others may be more ubiquitous.

Analysis of GR mRNA expression in the lateral OFC, in the context of sequence variation in the NR3C1 gene, did not reveal any relationship of NR3C1 genotype to lateral OFC gene expression. This finding is in contrast to our previous observations in the DLPFC, in which the rs10052957 (Tth111l) and rs6190 (R23K) SNPs impacted expression of the GR-1B and GR-1C mRNA transcript variants respectively [41]. Such polymorphisms may, therefore, impact NR3C1 gene expression in an anatomically specific manner. This effect could be mediated by brain region-specific transcription factors, which may influence regional patterns of GR mRNA transcript variant expression [59]. Such DLPFC and lateral OFC region-specific transcription factors could differentially interact with NR3C1 polymorphisms, manifesting effects of genotype in the DLPFC but not the lateral OFC. Alternatively, the effect of genotype on GR gene expression may represent a gene × environment interaction, which is not evident in the lateral OFC because this region may be less susceptible than the DLPFC to the effects of stress or other environmental influences. The effects of NR3C1 genotype on GRα protein which was previously observed in the DLPFC [41] were not seen in the lateral OFC in this study. The mechanisms by which genotype may impact GRα protein isoform levels in a brain region-specific manner remain to be elucidated.

In this study, our observations of both similarities (at the GRα protein level) and differences (at the GR mRNA level) between schizophrenia and bipolar disorder are consistent with the similarities and differences between the two illnesses more generally. Bipolar disorder and schizophrenia share similar psychotic symptoms, genetic and environmental risk factors, HPA axis abnormalities and some similar neuropathological changes [19,23-25,60,61]. However, the two illnesses differ from each other in the extent of their affective symptoms, cognitive disturbances and in other neuropathological changes [62-66]. Our findings support the notion that a complex relationship exists between schizophrenia and bipolar disorder at a neurobiological level.

Overall, we identified abnormal GR mRNA and GRα protein expression in the lateral OFC in schizophrenia and bipolar disorder. Depending on the functional properties of the GRα-D1 isoform, these changes have the capacity to impact cellular stress responses of neurons within the lateral OFC. It has been shown that the stress hormone cortisol, acting through GR in other brain regions, can impact glutamatergic and GABAergic neurotransmitter signalling [67-72], and in excess can cause neuronal loss and impair brain function [73-75]. It is plausible, therefore, that high cortisol levels in some individuals with schizophrenia and bipolar disorder may act via glutamatergic and GABAergic mechanisms to contribute to abnormalities (structural and functional) in the lateral OFC network, in a process either mediated by, or resulting in, GR mRNA and GRα protein dysregulation in the lateral OFC. The possible roles of stress and GR dysregulation in the pathophysiology of schizophrenia and bipolar disorder, potentially through interaction with the glutamatergic and GABAergic neurotransmitter systems in the prefrontal cortex, warrant further study.

## Conclusions

In this study, we provide evidence of GR mRNA and GRα protein isoform abnormalities in the lateral OFC in bipolar disorder and schizophrenia. These findings particularly highlight the potential importance of the functional GRα-D1 isoform in psychotic mental illness. Directions for future studies may include investigation of mechanisms through which GRα-D1 dysregulation may impact the function of the prefrontal cortex. Ultimately, understanding the molecular basis of HPA axis abnormalities in schizophrenia and bipolar disorder may enable therapeutic interventions aimed at lowering stress-related risk for psychosis.

## Competing interests

The authors declare that they have no competing interests.

## Authors’ contributions

DS contributed to the design of the study, carried out all experiments, analysed the data and wrote the manuscript. MJW developed the post-mortem tissue cohort, provided human neuroanatomical expertise, performed RNA and protein extractions and contributed to analysis of demographic data. JMF oversaw analysis of the genotype data and edited the manuscript. CSW conceived of the study and participated in its design and coordination, contributed to data analysis and edited the manuscript. All authors read and approved the final manuscript.

## Pre-publication history

The pre-publication history for this paper can be accessed here:

http://www.biomedcentral.com/1471-244X/12/84/prepub
